# Laparoscopic-assisted subtotal colectomy cecal-rectal anastomosis for redundant colon-associated slow-transit constipation: a single-center retrospective case series with long-term follow-up

**DOI:** 10.3389/fmed.2026.1839442

**Published:** 2026-07-06

**Authors:** Wen-Na Liu, Zheng-Hong Jiang, Qian-Qiu Zhang, Li-Hao Deng

**Affiliations:** 1 Department of Laboratory Medicine, Sichuan Provincial People’s Hospital, School of Medicine, University of Electronic Science and Technology of China, Chengdu, China; 2 Department of Gastrointestinal Hernia and Abdominal Wall Surgery, Affiliated Hospital of Chengdu University, Chengdu, Sichuan, China

**Keywords:** cecal-rectal anastomosis, laparoscopy, redundant colon, slow transit constipation, subtotal colectomy

## Abstract

**Background:**

Redundant colon represents a common anatomical substrate underlying slow transit constipation (STC). Although total colectomy with ileorectal anastomosis (TAC-IRA) reliably alleviates constipation, it is frequently associated with debilitating postoperative diarrhea. Subtotal colectomy cecal-rectal anastomosis (SCC-CRA) has emerged as a bowel-preserving alternative hypothesized to reduce diarrheal complications. This study aimed to evaluate the short- and long-term efficacy of laparoscopic-assisted SCC-CRA in patients with redundant colon.

**Methods:**

A retrospective analysis was conducted on 34 patients with redundant colon who underwent laparoscopic-assisted SCC-CRA between January 2016 and June 2025 at Chengdu University Affiliated Hospital. Clinical outcomes, including weekly bowel frequency, straining symptoms, and stool consistency (Bristol Stool Form Scale), were assessed preoperatively and at 8 months postoperatively. Long-term follow-up was performed at 2, 4, and 6 years after surgery.

**Results:**

All 34 patients (9 male, 25 female) successfully underwent the procedure without conversion to laparotomy. At 8 months postoperatively, all patients achieved more than three bowel movements per week (mean, 1–2 times/day), compared with 32 patients (94.1% [95% CI: 76–98%]) who had fewer than three movements per week preoperatively. Straining during defecation resolved completely, decreasing from 32 patients (94.1% [95% CI: 76–98%]) to none, and hard or lumpy stools (Bristol type 1–2) were eliminated, decreasing from 31 patients (91.2% [95% CI: 76–98%]) to none. Patients have a low reported incidence of diarrhea. Bowel frequency gradually normalized over time, declining from a peak of 4.8 ± 1.5 times/day at 3 months to 3.7 ± 1.3 times/day at 6 months. Long-term follow-up was available for 28 out of 34 patients (82.4%) at 2 years, 22 out of 34 patients (64.7%) at 4 years, and 15 out of 34 patients (44.1%) at 6 years, no symptomatic recurrence among available follow-ups.

**Conclusion:**

Laparoscopic-assisted subtotal colectomy cecal-rectal anastomosis is feasible with acceptable short-term outcomes as a surgical option for patients with redundant colon, providing significant and durable relief of constipation symptoms with a low risk of postoperative diarrhea. Both short-term and long-term outcomes are favorable.

## 1 Introduction

Chronic constipation represents one of the most prevalent gastrointestinal disorders worldwide (1, 2), with an estimated prevalence of 10–15% among adults in the United States (3–5). The condition substantially impairs patients’ quality of life (6) and imposes a considerable burden on healthcare systems (7, 8). Chronic constipation is classified into slow transit constipation, outlet obstruction constipation, mixed constipation, and normal transit constipation. Among these, slow transit constipation (STC) caused by redundant colon is one of the many subtypes of chronic constipation (9, 10), which accounts for 15–42% of cases and is characterized by impaired colonic motor activity leading to delayed transit of intraluminal contents (11–13). The etiology of STC is multifactorial, encompassing demographic, socioeconomic, lifestyle, and psychological factors. When conservative management fails and symptoms severely compromise daily functioning, surgical intervention becomes the treatment of last resort (14).

Surgical treatment for STC has a history spanning more than a century; however, the optimal procedure remains controversial owing to the heterogeneous clinical presentation and complex pathophysiology of the condition. Total colectomy with ileorectal anastomosis (TAC-IRA) is currently recommended by the American Society of Colon and Rectal Surgeons for patients with refractory STC (15–17). Although TAC-IRA reliably relieves constipation, it is frequently associated with postoperative diarrhea (18–20), which may compromise quality of life and lead to patient dissatisfaction (21, 22).

In contrast, subtotal colectomy cecal-rectal anastomosis (SCC-CRA) has emerged as an alternative that preserves the cecum, ileocecal valve, and a segment of the ascending colon (23–25). This approach is theoretically advantageous because it maintains water and electrolyte absorption while providing a reservoir function, thereby reducing the risk of postoperative diarrhea. The preservation of the ileocecal valve also allows for the retention of colonic bacterial flora, which metabolizes undigested starch and produces short-chain fatty acids, potentially contributing to normal stool consistency. Several studies have reported favorable outcomes with SCC-CRA, demonstrating comparable constipation relief to TAC-IRA with a significantly lower incidence of diarrhea (26, 27). Nevertheless, some investigators have raised concerns regarding a potentially increased risk of recurrent constipation with this procedure.

Despite these potential advantages, the available evidence supporting SCC-CRA for redundant colon is derived predominantly from retrospective, small-sample studies with low-quality evidence, and long-term outcome data remain limited. Adopting prospective, multicenter and protocol-driven designs alongside digitally assisted assessments will facilitate the advancement of modern minimally invasive colorectal research. Prospective multicenter validation studies should be conducted, and the criteria for outcome measurement need to be further standardized (28). The Slow Transit Obstipated Constipation Prospective Study (STOPS) trial is the first multicenter randomized controlled trial to compare TAC-IRA with SCC-CRA. The ongoing STOPS trial may provide higher-level evidence in the future (29).

This study aims to evaluate the short- and long-term efficacy of laparoscopic-assisted subtotal colectomy cecal-rectal anastomosis in patients with redundant colon, with the goal of providing evidence to guide surgical decision-making for this challenging patient population.

## 2 Materials and methods

### 2.1 Study design and patients

This retrospective study included 34 patients with redundant colon who underwent laparoscopic-assisted subtotal colectomy cecal-rectal anastomosis between January 2016 and June 2025 at the Department of Gastrointestinal Surgery, Chengdu University Affiliated Hospital. The study protocol was approved by the Institutional Ethics Committee of Chengdu University Affiliated Hospital. All data were obtained from the Department of Gastrointestinal Surgery, Affiliated Hospital of Chengdu University. Study protocol number: V1.0, 2026.03.23; approval number: PJ2026-034-03. All patient information was obtained with the informed consent of the patient. Clinically, defecography, colonic transit testing, dynamic pelvic magnetic resonance imaging (MRI), and anorectal manometry are routine examinations for constipation that facilitate its classification and etiological diagnosis (30, 31). All enrolled patients completed comprehensive preoperative evaluations to characterize the disease and exclude pelvic floor dysfunction. Anorectal manometry was performed to measure anal resting pressure, maximum anal squeeze pressure, the rectoanal inhibitory reflex (RAIR), pelvic floor coordination during simulated defecation, and rectal sensory function using the balloon distension test (32). All enrolled patients exhibited significantly elevated rectal sensory thresholds, with all other manometric parameters being normal. The radiopaque marker colonic transit test was performed, with abdominal X-rays taken at 6, 24, 48, and 72 h after marker administration. Delayed colonic transit was defined as failure to expel 80% of the markers within 72 h (4); all patients had positive results. Defecography, including barium X-ray defecography (BD) and MRI defecography (MRD), is a crucial examination for the diagnosis and classification of outlet obstructive constipation (33). It dynamically visualizes morphological abnormalities such as rectocele, internal rectal prolapse, and pelvic floor spasm during defecation. BD is simple and is the preferred modality for evaluating outlet obstructive constipation (34), whereas MRD offers the advantages of no radiation exposure and superior soft-tissue resolution (35). In this study, all patients showed normal anorectal and pelvic floor anatomy, normal defecatory dynamics and coordination, and no signs of outlet obstruction on defecography. The balloon expulsion test, a simple screening tool for assessing rectal emptying and pelvic floor coordination (36, 37), yielded normal results in all enrolled patients. Barium enema was used to diagnose redundant colon; Classification of redundant colon was defined as follows. Type I (sigmoid redundancy): sigmoid colon length >40 cm, with increased loops, a tortuous course, the upper margin of the loops extending above the iliac crest line, and markedly increased mobility. Type II (transverse colon redundancy): obvious ptosis of the transverse colon with its lowest point below the iliac crest line, downward displacement of the hepatic and splenic flexures, and lax, tortuous bowel. Type III (flexural redundancy): formation of redundant loops and folding at the hepatic and/or splenic flexures, resulting in localized elongation of the colonic segment. Total dolichocolon was defined as the coexistence of the above findings at multiple sites, characterized by diffuse colonic elongation, extensive tortuosity, and decreased colonic tone (38, 39). Total colonic redundancy was defined by diffuse elongation, extensive tortuous folding, decreased colonic tone, and multiple loop formation. Taken together with the normal findings on anorectal manometry, defecography, and balloon expulsion testing, pelvic floor dyssynergia was strictly excluded in all enrolled patients.

#### 2.1.1 Inclusion criteria

1 Diagnosis of STC according to Rome IV criteria, excluding outlet obstruction constipation, megacolon, organic constipation, drug-induced constipation, and irritable bowel syndrome.
2 Confirmed redundant colon by barium enema.
3 Failure of at least 2 years of systematic non-surgical treatment.
4 Severe constipation symptoms affecting daily life with strong willingness for surgery.
5 Prolonged colonic transit time, with normal findings on defecography, anorectal manometry, balloon expulsion test, pelvic floor electromyography, and anal endoscopic ultrasound (excluding outlet obstruction).
6 No psychiatric disorders or surgical contraindications.
7 All patients underwent laparoscopic-assisted subtotal colectomy cecal-rectal anastomosis.

#### 2.1.2 Exclusion criteria

1 No definitive diagnosis of redundant colon on barium enema.
2 STC combined with outlet obstruction constipation or megacolon.
3 Organic or drug-induced constipation.
4 Emergency surgery for colonic volvulus, obstruction, or perforation.
5 Pathological findings inconsistent with redundant colon.
6 History of abdominal or pelvic surgery or trauma.
7 Incomplete clinical data or loss to follow-up.

### 2.2 Surgical technique

**Figure 1:**
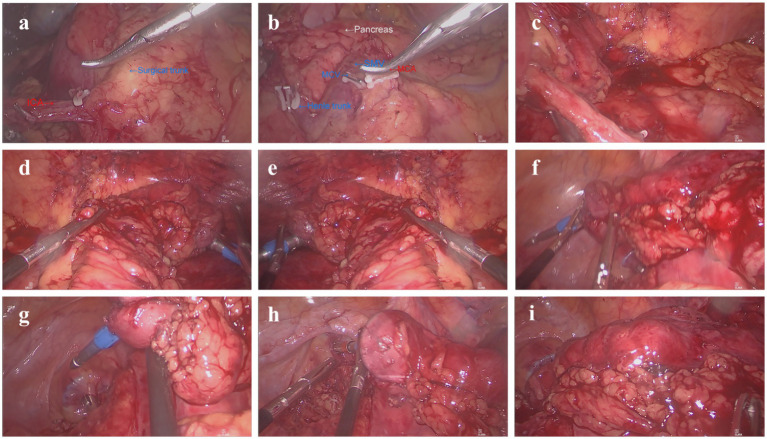
Key steps of laparoscopic-assisted subtotal colectomy with cecal-rectal anastomosis. **(a)** Preservation of the ileocolic artery. **(b)** Skeletonized superior mesenteric artery. **(c–h)** Rotation of the retained 5-cm ascending colon 180° counterclockwise into the pelvic cavity. **(i)** Completed Isoperistaltic colorectal anastomosis.

All operations were performed by the same surgical team under general anesthesia. The colon was mobilized laparoscopically with preservation of the ileocolic vascular pedicle and its branches. A 4–5 cm incision was made at the right lower abdominal trocar site for specimen extraction. After specimen removal, the bowel was transected approximately 3–5 cm from the ileocecal junction, and the anvil of a 29 mm circular stapler was inserted into the distal ascending colon. The cecum was then placed into the pelvic cavity counterclockwise without torsion. After appendectomy, a circular stapler was introduced transanally to create an isoperistaltic colorectal anastomosis between the cecum and the rectal stump, with meticulous attention to achieving a tension-free anastomosis. Subsequently, the colorectal anastomosis was reinforced using 3–0 barbed sutures. All mesenteric defects were closed to prevent postoperative internal hernia and colonic torsion (Figure 1).

### 2.3 Outcome measures

#### 2.3.1 Primary outcomes

- Weekly bowel movements (categorized as <3 vs. ≥3 times/week).
- Presence of straining during defecation.
- Stool consistency assessed by the Bristol Stool Scale (type 1–2: hard/lumpy; type 4–5: normal).

#### 2.3.2 Secondary outcomes

- Bowel frequency and stool consistency at 3 and 6 months postoperatively.
- Long-term outcomes at 2, 4, and 6 years postoperatively.

### 2.4 Follow-up

Patients were followed up via outpatient visits and telephone interviews at 3 months, 6 months, 8 months, 2 years, 4 years, and 6 years postoperatively. Data on bowel habits, straining symptoms, stool consistency, and complications were collected.

### 2.5 Statistical analysis

Statistical analysis was performed using SPSS version 25.0 (IBM Corp. Armonk, NY, United States). Continuous data are presented as mean ± standard deviation or median (interquartile range) based on distribution. The paired *t*-test or Wilcoxon signed-rank test was used to compare continuous variables before and after surgery. Categorical variables were analyzed using McNemar’s test or the chi-square test. A two-tailed *p*-value <0.05 was considered statistically significant.

## 3 Results

### 3.1 Patient characteristics

A total of 34 patients with redundant colon were included in the present analysis. The cohort comprised 9 male (26.5%) and 25 female (73.5%) patients, with a mean age of 48.6 ± 12.3 years (range, 22–71 years). Regarding the anatomical classification of colonic redundancy, 8 patients (23.5%) presented with type I, 22 patients (64.7%) with type II, and 4 patients (11.8%) with type III. No patient presented with comorbidities that would contraindicate surgical intervention. Detailed baseline characteristics are summarized in Table 1.

**Table 1:** Baseline characteristics of patients with redundant colon (*N* = 34).

Characteristic	Value
Sex, *n* (%)	
Male	9 (26.5)
Female	25 (73.5)
Age, years	
Mean ± SD	48.6 ± 12.3
Range	22–71
Colonic redundancy classification, *n* (%)	
Type I	8 (23.5)
Type II	22 (64.7)
Type III	4 (11.8)
Comorbidities precluding surgery	0 (0)

### 3.2 Surgical outcomes

**Figure 2:**
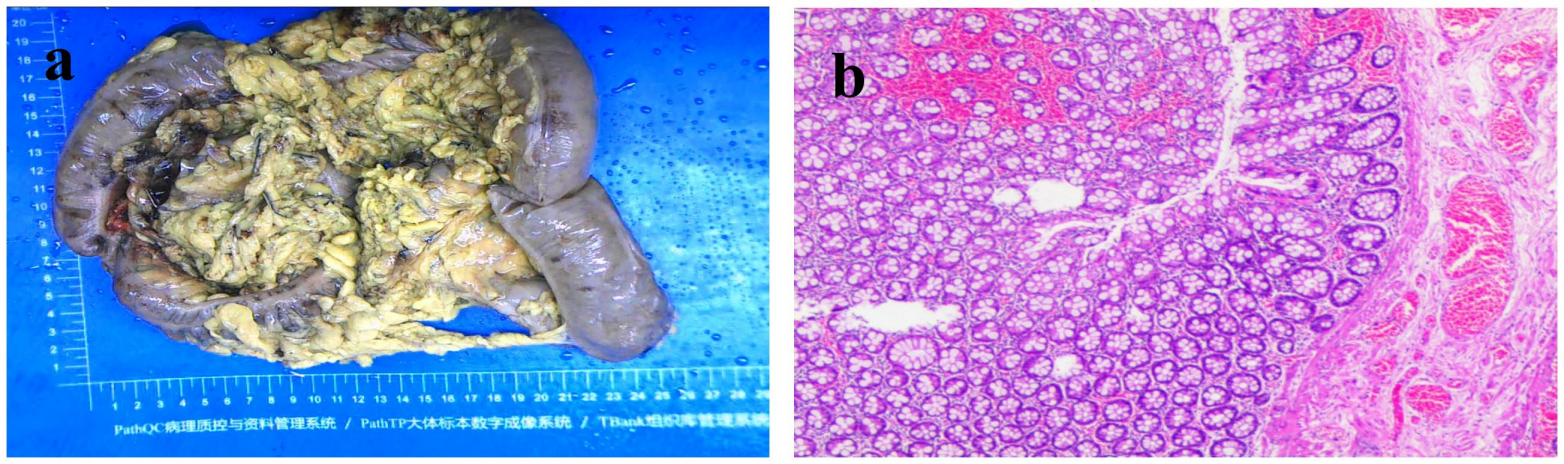
Surgical specimen and pathological findings. **(a)** Gross appearance of the resected colon specimen. **(b)** Postoperative histopathology confirmed the diagnosis consistent with slow transit constipation.

All 34 patients successfully underwent laparoscopic-assisted subtotal colectomy with cecal-rectal anastomosis without conversion to open surgery. The mean operative time was 285.6 ± 45.2 min, and the mean estimated blood loss was 98.5 ± 42.3 mL. Gross examination of the resected colon specimen confirmed the presence of colonic redundancy, and postoperative histopathological findings were consistent with the diagnosis of slow transit constipation (Figure 2).

### 3.3 Improvement in constipation symptoms

**Figure 3:**
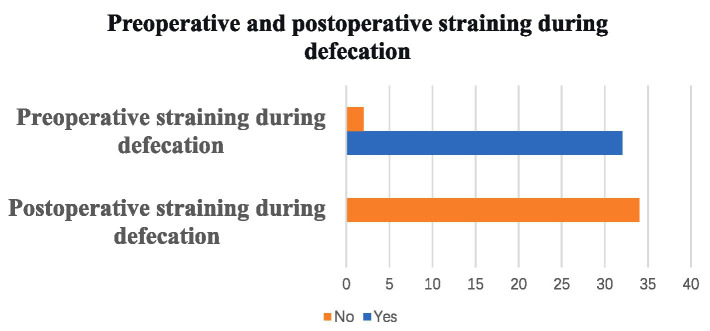
Preoperative vs. postoperative straining symptoms. Bar chart: Preoperative: 94.1% with straining; Postoperative: 0% with straining.

**Figure 4:**
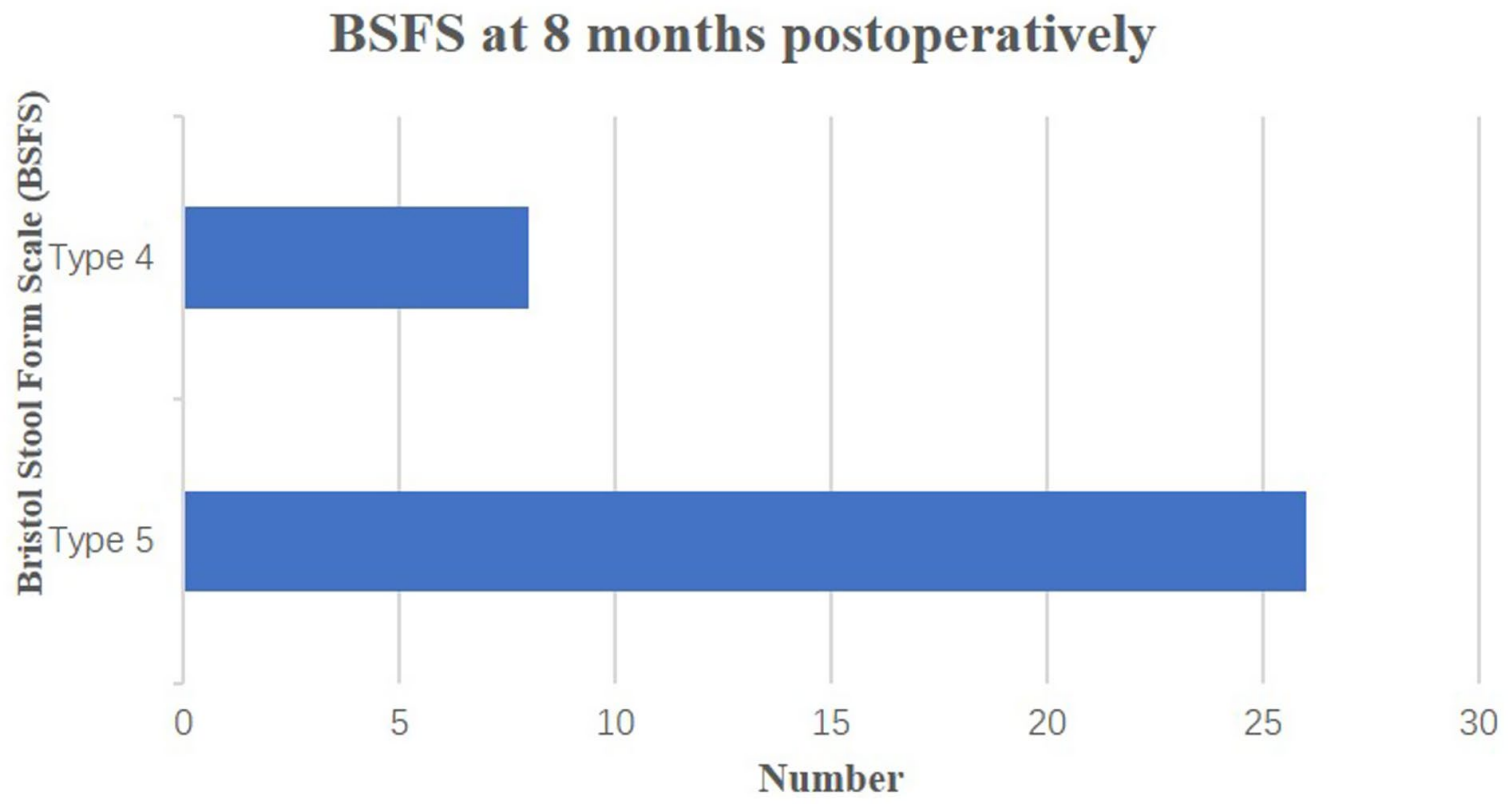
Bristol stool scale distribution at 8 months postoperatively. Bar chart: 100% Bristol type 4–5.

At 8 months postoperatively, all 34 patients (100%) achieved more than three bowel movements per week, with a mean frequency of one to two times per day, compared with 32 patients (94.1% [95% CI: 76–98%]) who had fewer than three bowel movements per week preoperatively. Straining during defecation resolved completely, decreasing from 32 patients (94.1% [95% CI: 76–98%]) preoperatively to none postoperatively (Figure 3). Hard or lumpy stools (Bristol type 1–2) were present in 31 patients (91.2% [95% CI: 76–98%]) before surgery, whereas no patient reported this consistency at 8 months postoperatively. By the 8-month follow-up, all patients had achieved normal stool consistency (Bristol type 4–5) (Figure 4). Detailed comparisons of constipation symptoms before and after surgery are shown in Table 2.

**Table 2:** Comparison of constipation symptoms before and after surgery.

Variable	Preoperative (*n* = 34)	Postoperative (8 months) (*n* = 34)	*p*-value
Weekly bowel movements (<3 times)	32 (94.1%)	0 (0%)	<0.001
Straining during defecation	32 (94.1%)	0 (0%)	<0.001
Hard or lumpy stools (Bristol type 1–2)	31 (91.2%)	0 (0%)	<0.001

Data are presented as *n* (%). Statistical analysis: McNemar’s test.

**Figure 5:**
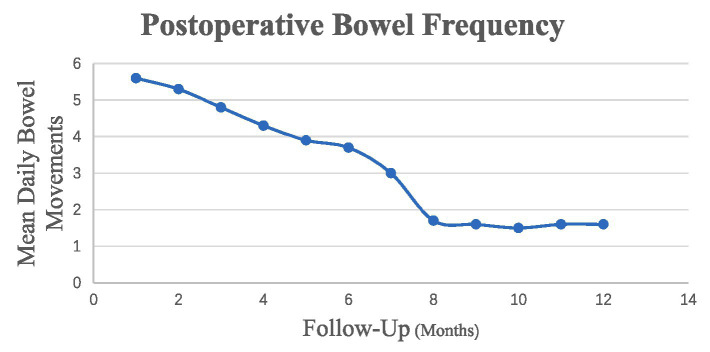
Trend of bowel movements during the first 12 months postoperatively. The graph shows the change in weekly bowel movement frequency following laparoscopic-assisted subtotal colectomy with cecal-rectal anastomosis. A gradual increase in stool frequency was observed, with stabilization achieved by 8 months after surgery.

During the first 12 months after surgery, bowel movement frequency gradually increased and stabilized by 8 months postoperatively (Figure 5).

### 3.4 Longitudinal follow-up

During the early postoperative period, bowel frequency peaked at 3 months, with a mean of 4.8 ± 1.5 times per day and loose stool consistency. By 6 months, the mean frequency decreased to 3.7 ± 1.3 times per day, with stools becoming soft or fragmented (Bristol type 5–6). At 8 months postoperatively, bowel frequency stabilized at one to two times per day with normal formed stools (Bristol type 4–5). Long-term follow-up was available for 28 patients (82.4%) at 2 years, 22 patients (64.7%) at 4 years, and 15 patients (44.1%) at 6 years. At all-time points, patients maintained a bowel frequency of one to two times per day with normal stool consistency (Bristol type 4–5) and absence of straining symptoms. No case of constipation recurrence was observed during the follow-up period. Detailed longitudinal data are summarized in Table 3.

**Table 3:** Longitudinal follow-up of bowel frequency and stool consistency.

Time point	*n*	Bowel frequency (times/day)	Stool consistency (Bristol type)
Preoperative	34	<3/week	Type 1–2 (hard/lumpy)
3 months	34	4.8 ± 1.5	Loose/porridge-like
6 months	34	3.7 ± 1.3	Type5-6 (soft/fragmented)
8 months	34	1–2	Type 4–5 (formed)
2 years	28	1–2	Type 4–5 (formed)
4 years	22	1–2	Type 4–5 (formed)
6 years	15	1–2	Type 4–5 (formed)

Data are presented as mean ± standard deviation or as range.

In addition to primary symptomatic indicators, the validated Wexner Constipation Score was adopted to quantitatively evaluate overall constipation severity. The mean preoperative total score was 18.6 ± 4.2, indicating moderate to severe constipation in all enrolled patients. The Wexner score decreased significantly at all postoperative follow-up time points. At 8 months after surgery, the mean score dropped to 0.9 ± 1.1, suggesting nearly complete relief of constipation symptoms. During long-term follow-up (2, 4 and 6 years), the Wexner score remained at a low level without obvious fluctuation, demonstrating sustained improvement of defecation function. No patient required laxatives, enema or manual assistance for defecation after 8 months postoperatively. None of the patients required repeat surgical intervention (Table 4).

**Table 4:** Changes in Wexner constipation score at different follow-up time points.

Time point	*n*	Wexner total score (x ± s)
Preoperative	34	18.6 ± 4.2
Postoperative 3 months	34	5.3 ± 2.1*
Postoperative 6 months	34	2.8 ± 1.6*
Postoperative 8 months	34	0.9 ± 1.1*
Postoperative 2 years	28	0.8 ± 1.0*
Postoperative 4 years	22	0.7 ± 0.9*
Postoperative 6 years	15	0.6 ± 0.8*

*p < 0.001, compared with preoperative score.

### 3.5 Complications

Postoperative complications were assessed and stratified according to the Clavien-Dindo classification system. A total of 26 patients presented with Grade I complications, including postoperative pain, vomiting, and electrolyte disorders. Additionally, wound incision infection was observed in four patients, which was also classified as Grade I. Two patients developed postoperative intestinal obstruction corresponding to Grade II complications, and two patients suffered from severe anastomotic leakage, categorized as Grade IIIb complications. All patients with Grade I and II intestinal obstruction achieved complete recovery after conservative management. Similarly, the two patients with Grade IIIb anastomotic leakage recovered uneventfully following adequate drainage and conservative treatment. No Grade V mortality occurred within 30 days postoperatively. Regarding postoperative recovery indicators, the median hospital stay was 7 days (interquartile range, 5–10 days). The median time to first liquid diet resumption, first flatus, and first defecation was 2 days, 3 days, and 4 days, respectively. Limited available data suggest that robotic surgery may be associated with lower mortality and readmission rates compared with laparoscopic surgery in elderly patients (40).

## 4 Discussion

Slow transit constipation (STC), characterized by delayed colonic transit, is one of the most common functional gastrointestinal disorders (4, 41), though its pathogenesis remains incompletely understood (42). While most patients respond to conservative medical management, a subset with refractory STC who fail prolonged nonoperative treatment may require surgical intervention (14, 43, 44). For patients who do not respond to nonoperative management, surgery offers an effective therapeutic option. Currently, the most commonly employed surgical procedures for STC are total colectomy with ileorectal anastomosis (TAC-IRA) (45–47) and subtotal colectomy cecal-rectal anastomosis (SCC-CRA) (48–50); however, no standardized surgical approach has been established. Segmental colectomy has been largely abandoned due to failure rates as high as 100% (21, 22, 51). TAC-IRA achieves clinical improvement rates approaching 100% and is widely recommended by many colorectal surgeons. Nevertheless, this procedure is associated with a high incidence of postoperative diarrhea, abdominal pain, and fecal incontinence, which adversely affect quality of life, with patients reporting significantly lower quality of life compared to the general population (20, 52–54). SCC-CRA is another commonly used surgical option for STC. By preserving the ileocecal junction, this procedure may offer theoretical advantages. Subtotal colectomy with preservation of the ileocecal valve can be performed either as cecal-rectal anastomosis or as Ile sigmoid anastomosis with preservation of the distal sigmoid colon. Both techniques effectively improve bowel frequency in patients with STC, with reported overall efficacy and patient satisfaction rates ranging from 39 to 100% (55). Deloyers procedure which preserves the ileocecal valve, avoids the need for total colectomy and ileorectal anastomosis, and has been associated with lower anastomotic leak rates compared to TAC-IRA. Preservation of the ileocecal valve may reduce the severity of postoperative diarrhea. The ileocecal region plays important roles in preventing reflux of colonic contents, maintaining the spatial structure of the gut microbiota, and regulating the intestinal immune and inflammatory microenvironment. Preservation of this region may facilitate postoperative recovery of gastrointestinal function, alleviate postoperative intestinal inflammation, and help maintain gut microbiota and bile acid homeostasis (56). Therefore, surgical strategies that preserve the ileocecal region hold clinical relevance (25, 57).

Among ileocecal-preserving subtotal colectomy procedures, two main anastomotic configurations exist: antiperistaltic and isoperistaltic configurations. In the antiperistaltic technique, the anvil of a circular stapler is inserted via the appendiceal stump after appendectomy, allowing direct anastomosis with the rectal stump without the need for ileocecal rotation. This approach is technically simpler and may offer advantages in terms of quality of life. However, from an anatomical and physiological perspective, the antiperistaltic configuration alters the intrinsic direction of bowel motility and may increase the risk of postoperative complications such as abdominal bloating and intestinal obstruction (58). In contrast, the isoperistaltic (Deloyers) technique involves rotating the ileocecal segment to preserve the natural direction of peristalsis. The technical key to this procedure is the preservation of approximately 5 cm of the cecum along with the ileocecal valve, which may help mitigate the frequency of postoperative diarrhea and has minimal impact on daily activities. The Deloyers technique requires complete mobilization of the right colon and its mesentery with preservation of the ileocolic vessels. A critical step is the 180° counterclockwise rotation of the ileocecal segment, resulting in a mirrored orientation. This allows the ileum to lie naturally along the right paracolic gutter. Securing the mesenteric edge of the preserved ileocecal segment to the retroperitoneum and pelvic wall with barbed sutures can effectively prevent internal herniation and reduce the risk of serious complications such as postoperative intestinal necrosis (58). Evidence shows that digestive tract reconstruction using a colonic J-pouch helps reduce bowel frequency and fecal urgency, and lower the proportion of patients who require laxatives for diarrhea. This technique yields long-term outcomes comparable to straight coloanal anastomosis and superior to side-to-end anastomosis (59).

Several limitations should be acknowledged. First, this was a single-center retrospective study with a relatively small sample size (*n* = 34), which may limit generalizability. Second, the absence of a concurrent control group (e.g., TAC-IRA) precludes direct comparison of surgical techniques. Third, the loss to follow-up at 6 years (44.1% retention) may introduce attrition bias.

The postoperative recovery trajectory observed in this study is consistent with previous reports. The initial increase in bowel frequency (4.8 ± 1.5 times/day at 3 months) reflects the adaptation period of the residual colon. With dietary guidance and gradual colonic adaptation, bowel frequency normalized by 8 months, and stool consistency improved from loose to formed. This pattern highlights the importance of patient education during the early postoperative period.

The Wexner Constipation Score, a classic quantitative instrument for evaluating functional defecatory disorders, further validates the clinical efficacy of laparoscopic-assisted subtotal colectomy with cecal-rectal anastomosis. The continuous decline and long-term stability of scores confirm that this surgical procedure can comprehensively relieve constipation-related symptoms, rather than merely improving single indicators such as bowel frequency and stool consistency. Combined with the finding that no patients in this cohort developed postoperative diarrhea, the results indicate that this technique achieves a favorable balance between regulating defecation frequency and preserving intestinal function, with potentially satisfactory long-term functional outcomes.

Despite these limitations, this study provides valuable evidence supporting the efficacy of laparoscopic-assisted SCC-CRA for redundant colon. Future multicenter, prospective, randomized controlled trials are needed to compare SCC-CRA with TAC-IRA and to establish standardized patient selection criteria.

## 5 Conclusion

Laparoscopic-assisted Subtotal colectomy cecal-rectal anastomosis may be feasible and associated with symptomatic improvement in carefully selected patients. This procedure represents a favorable surgical option for slow transit constipation caused by colonic redundancy.

## Data Availability

The datasets generated and analysed during the current study are not publicly available due to institutional privacy restrictions, but are available from the corresponding author upon reasonable request.
